# Examining the Effects of PrEP Use on Sexual Behaviors and Sexually Transmitted Infections Among Chinese Men who have Sex with Men: A Cross-Sectional Study

**DOI:** 10.1007/s10461-024-04398-9

**Published:** 2024-07-27

**Authors:** Bingyang She, Fang Lu, Rui Zhao, Siqi Lin, Jiajun Sun, Shiyi He, Yi Liu, Shu Su, Lei Zhang

**Affiliations:** 1 China-Australia Joint Research Center for Infectious Diseases, School of Public Health, Jiaotong University Health Science Center, Xi’an, Shaanxi 710061 China; 2 Melbourne Sexual Health Centre, Alfred Health, Melbourne, Australia; 3 Central Clinical School, Faculty of Medicine, Nursing and Health Sciences, Monash University, Melbourne, VIC Australia; 4 Department of Epidemiology and Biostatistics, The Second Affiliated Hospital of Chongqing Medical University, Chongqing, China

**Keywords:** PrEP willingness, PrEP usage, STI infections, MSM, Shannon diversity index

## Abstract

Men who have sex with men (MSM) is a high-risk population for HIV and sexually transmitted infections (STIs). Pre-exposure prophylaxis (PrEP) is effective in HIV prevention. This study aims to examine the differences in sexual behaviors, STI prevalence and HIV/STI testing across subgroups of MSM with various PrEP use. Data were collected via a cross-sectional survey in an MSM community in Xi’an, Shaanxi, from 2022.01 to 2022.09. Participants were categorized as ‘PrEP-naïve and unwilling to use’, ‘PrEP-naïve but willing to use’, and ‘current or former PrEP users’. Shannon index was used to assess sexual act diversity and multivariate logistic regression analyzed factors associated with PrEP use. Of the 1,131 MSM participants, 23.52% were PrEP-naïve and unwilling, 64.98% were PrEP-naïve but willing, and 11.49% were current or former PrEP users. The PrEP-naïve but willing group had the highest recent STI testing rates at 73.06% and showed greater sexual act diversity (Shannon index 1.61). This group also had the highest syphilis rates (7.49% vs. 6.47% and2.54%, *p* < 0.01). Younger age (18–30: OR = 0.39 (0.18–0.85); 31–40: OR = 0.43 (0.20–0.96)) and lower education (high school/vocational: OR = 0.15 (0.04–0.58); associate degree: OR = 0.21 (0.06–0.71)) were factors that negatively influenced PrEP use. Current or former PrEP users had the highest oropharyngeal gonorrhea (14.39% vs. 9.68% and 5.80%, *p* < 0.01) and overall gonorrhea rates (20.86% vs. 17.17% and 8.37%, *p* < 0.001). ‘PrEP-naïve but willing’ participants consistently demonstrated high-risk sexual behavior, increased STI testing, and more diverse sexual acts, whereas PrEP users had the highest STI prevalence.

## Introduction

According to the World Health Organization, 39 million people worldwide were living with HIV in 2022 [1–3]. As of October 2021, China reported 1.14 million people living with HIV/AIDS, an 8.3% increase from 2020, with 111,000 new infections [4]. HIV prevalence among MSM, a major high-risk group in China, rose from 4.5% in 2011 to 8% in 2019 [5, 6]. From 2010 to 2018, newly diagnosed HIV infections increased annually among MSM, particularly in western China [7–9]. Between 2016 and 2020, the HIV infection rate among student men who have sex with men increased from 5.2–8.3% [10].

Pre-exposure prophylaxis (PrEP) has been shown to be highly effective in reducing the risk of HIV transmission in high-risk populations [11–14]. For the MSM population, its efficacy in preventing HIV can reach up to 96% if the adherence rate is optimal [15, 16,65, 66]. In light of this, WHO has recommended the use of PrEP for MSM in 2014 [12]. In response to this recommendation, a pilot of PrEP was initiated in China in 2019, during which the National Medical Products Administration approved the use of emtricitabine/tenofovir disoproxil fumarate (brand name: Shufatai®) as PrEP [17]. There are plans to pilot it in several cities [18–20,64, 67].

Successful prevention strategy relies on the acceptance and adoption of the target population. Existing studies reported that PrEP usage varies across countries and regions, which may be influenced by a variety of factors such as socio-demographics, culture, economy and institutional frameworks [21–23]. Understanding the characteristics and facilitating factors behind PrEP uptake can maximize its benefits. While numerous studies have explored how economic status, knowledge, and attitudes affect PrEP usage among the MSM population, research focusing on the willingness to use and actual usage of PrEP is comparatively sparse [24–30]. Exploring the factors driving this disparity within the MSM community is crucial and warrants further investigation.

Sexually transmitted infections (STIs) significantly impact MSM, raising major health concerns [31]. Studies on PrEP’s use and its link to STIs have shown different results. Research is divided: the majority indicate PrEP users have a higher STI risk, while a minority see no difference from non-users [32, 33]. This debate may stem from high-risk groups within MSM that are not using PrEP. Earlier studies reveal MSM interested in PrEP often are at high risky behaviors, meeting its usage criteria [34]. When the proportion of those interested in PrEP varies within the non-using group, comparisons between PrEP users and non-users yield divergent outcomes. Additionally, this may overlook the fact that MSM using PrEP have a higher prevalence of STIs due to ‘risk compensation’, which involves reduced condom use and leads to an increased chance of contracting STIs [35–38]. Therefore, knowing if this group is also more prone to STIs can clarify earlier research discrepancies. Understanding those willing to use PrEP is crucial for crafting targeted, effective prevention for MSM.

Northwest China has been a high-risk area for HIV infections, with homosexual transmission among MSM playing a significant role in its spread [39, 40]. Xi’an is the most populous city in Northwest China, and it is estimated that there were 43,000 MSM by the end of 2020. This study aims to explore in depth the attitudes, willingness to use, and actual use of PrEP, as well as the prevalence of STIs among the MSM population with different PrEP usage in Xi’an, and identifies the underlying factors for their PrEP use. This study may provide strong guidance and support for future HIV/STI prevention efforts in the region.

## Methods

### Study Design and Participants

We conducted a cross-sectional study in the Xi’an Tongzhi community from January to September 2022 to investigate the usage of PrEP and its association with STIs prevalence among MSM in China. Participants were recruited through online and offline platforms, including social media, dating apps, and MSM community organizations. Eligibility criteria included being at least 18 years old, having had sexual relations with men in the past three months and either self-reporting a HIV-negative status or being unaware of their HIV status. We excluded individuals with self-reported severe mental illness, language barriers, intellectual disabilities, or inability to complete the survey as assessed by the investigator.

### Data Collection and Measurement

Participants completed an anonymous online questionnaire administered via Wenjuanxing (www.wjx.cn), collecting information on socio-demographic characteristics, PrEP usage and willingness, knowledge of HIV and PrEP, HIV infection risk perception, condom use during anal intercourse, and chemsex behaviors. The questionnaire was available in Chinese and administered via a secure online platform. The study utilized a combination of simple random sampling and snowball sampling methods, recruiting participants from MSM communities and venues in Xi’an. Participants underwent testing for common STIs, including Chlamydia trachomatis (CT), Neisseria gonorrhoeae (NG), HIV, and syphilis by investigator after completing the survey. In addition to the general tests, specific testing was conducted for NG and CT infections across different anatomical sites including the anus, oropharynx, and urethra. The nomenclature used for the specific tests were A-CT, A-NG for anal samples; O-CT, O-NG for oropharyngeal samples; and U-CT, U-NG for urethral samples, A stands for Anal, O for Oral, and U for Urethral. The CT and NG tests were performed using test kits (PCR-fluorescent probe method). CT and NG infections were tested by the Xi’an KingMed institution, while syphilis and HIV testing were conducted by the Xi’an Lianhu District CDC.

### Definition of Sex Acts, Sex Act Pairs and Sexual Diversity

We recorded the sequence of sexual activities in the participants’ most recent sexual encounter. The participants were allowed to fill up to 12 steps of sequence of sexual acts in the last sexual encounter. These steps encompassed a range of sexual behaviors, including kissing, insertive and receptive oral sex, insertive and receptive anal sex, rimming, being rimmed, masturbation (self, for a partner, by a partner). Participants were allowed to record repeated instances of the same sexual behavior within the sequence. The sequence was considered complete upon the conclusion of the sexual encounter. In a sexual episode, we treated consecutive repeated actions as a single act, such as ‘kissing-kissing’ simply as ‘kissing’. We also recorded chemsex as use of recreational drugs before and during the participant’s most recent sexual activity [41]. These recreational drugs include, but are not limited to, nitrite inhalants, Ketamine, Ecstasy (MDMA), and GHB/GBL. We defined a ‘sex act pair’ as the combination of two consecutive steps in the sequence involved different sexual behaviors. To calculate the number of such pairs, we use:

$$P\left(n,2\right)=\frac{n!}{\left(n-2\right)!}$$

Where $n$ is the total number of unique actions. We measured the diversity of sex act pairs with Shannon Index. The Shannon index, a measure commonly used in ecological studies to quantify species diversity, was repurposed in our study to represent the diversity index of the participants’ sexual behaviors. The formula for calculating the Shannon Index is as follows:

$$H=-{\sum }_{i=1}^{R}{P}_{i}\text{ln}\left({P}_{i}\right)$$

where $H$ represents the Shannon diversity index, $R$ is the total number of distinct sex act pairs observed, ${P}_{i}$ is the proportion of the ${\text{i}}^{\text{t}\text{h}}$ sex act pair relative to the total number of sex act pairs, and $ln$ denotes the natural logarithm. The Shannon Index values range from 0 to ${\infty }$, where 0 indicates no diversity and higher values indicate greater diversity [42].

### Statistical Analysis

Based on PrEP usage and willingness, we categorized the participants into three groups: (1) Those who have never used PrEP but are willing to use it(PrEP-naïve but willing); (2) Those who have never used PrEP and are unwilling to use it(PrEP-naïve and unwilling) ; and (3) Those who have used or currently use(current/former user). The data was described using descriptive statistics including interquartile ranges (IQR), frequencies, percentages. The Kruskal-Wallis H test was used to compare the sexual behavior diversity (Shannon Index) between groups. The chi-square test was employed to compare the demographic characteristics of the groups and the prevalence of STIs. A p-value of *p* < 0.05 was considered statistically significant. A two-step multiple logistic regression analysis was performed to determine the factors that influence PrEP usage. The dependent variable in this analysis was the PrEP usage status. A univariate logistic regression was conducted for each factor, with a significance level of *p* < 0.05. Numerical data were standardized to two decimal places for consistency, except where additional precision was necessary for statistical significance and accuracy. Significant factors were then included in a multivariate model to comprehensively evaluate their individual and combined effects on PrEP usage among the participants.

## Results

### Sociodemographic Characteristics of the Study Population

Of the initially recruited 1,337 potential participants, 206 were excluded from the study because of refusal to participate and sign the informed consent form (*N* = 13), missing test records (*N* = 164), and absence of PrEP usage intent data (*N* = 29), leaving a total of 1,131 participants for analysis. In the analysis population, the majority of participants were aged between 18 and 30 (38.28%) and lived in urban areas (54.20%). Most participants identified as homosexual (69.94%) and belonged to the Han ethnicity (96.20%). Over half of the participants had a high school education (53.50%), and 43.58% earned less than 3000 RMB per month. (Table 1)

**Table 1:** Demographic characteristics of participants and PrEP usage status

Category	Overall(N = 1131)	PrEP-naïve and unwilling group (N = 266)	PrEP-naïve but willing group (N = 735)	Current/former user group (N = 130)	Chi-Square	P-value
**Age group(years)**					12.24	0.016*
18–30	433 (38.28%)	74 (27.82%)	306 (41.63%)	53 (39.55%)		
31–40	390 (34.48%)	83 (31.20%)	262 (35.65%)	44 (32.84%)		
> 40	238 (21.04%)	68 (25.56%)	143 (19.46%)	28 (20.90%)		
**Current Residence**					82.78	<0.001**
Urban	613 (54.20%)	97 (36.47%)	432 (58.78%)	84 (62.69%)		
Town	279 (24.67%)	62 (23.31%)	190 (25.85%)	27 (20.15%)		
Rural	227 (20.07%)	103 (38.72%)	105 (14.29%)	19 (14.18%)		
**Sexual orientation**					4.32	0.364
Homosexuality	791 (69.94%)	190 (71.43%)	500 (68.03%)	100 (74.63%)		
Bisexual	312 (27.58%)	68 (25.56%)	215 (29.25%)	29 (21.64%)		
Others	17 (1.50%)	4 (1.50%)	12 (1.63%)	1 (0.75%)		
**Ethnicity**					5.83	0.054
Han ethnicity	1088 (96.20%)	259 (97.37%)	705 (95.92%)	123 (91.79%)		
Ethnic Minorities	32 (2.83%)	3 (1.13%)	22 (2.99%)	7 (5.22%)		
**Marital status (with female)**					4.83	0.305
Unmarried	690 (61.01%)	157 (59.02%)	443 (60.27%)	89 (66.42%)		
Married	341 (30.15%)	86 (32.33%)	220 (29.93%)	35 (26.12%)		
Others	89 (7.87%)	19 (7.14%)	64 (8.71%)	6 (4.48%)		
**Education level**					153.44	<0.001**
Junior high school and below	152 (13.45%)	89 (33.46%)	49 (6.67%)	14 (10.45%)		
High School (Secondary School)	605 (53.50%)	91 (34.21%)	496 (67.48%)	77 (57.46%)		
University (College)	289 (25.55%)	70 (26.32%)	194 (26.39%)	25 (18.66%)		
Graduated study and above	74 (6.54%)	12 (4.51%)	48 (6.53%)	14 (10.45%)		
**Monthly income**					166.82	<0.001**
Less than 3000 RMB	493 (43.58%)	91 (34.21%)	85 (11.56%)	26 (19.40%)		
3001-6000RMB	328 (29.01%)	121 (45.49%)	321 (43.67%)	50 (37.31%)		
6001-9000RMB	202 (17.87%)	40 (15.04%)	224 (30.48%)	44 (32.84%)		
Above 9000 RMB	97 (8.58%)	10 (3.76%)	77 (10.48%)	10 (7.46%)		

*Note* *: statistical significance at *p* < 0.05 level; **: higher level of statistical significance at *p* < 0.01

### PrEP Knowledge, HIV Risk Awareness, Condom Use, Chemsex and Shannon Index Among Three PrEP Usage Groups

Participants were categorized into three groups based on PrEP usage: PrEP-naïve but willing group(*N* = 735, 64.98%), PrEP-naïve and unwilling group (*N* = 266, 23.52%), and current/former user group(*N* = 130, 11.49%). PrEP-naïve but willing group were significantly younger than their counterparts, with a higher proportion falling into the 18–30 age range (41.63% vs. 27.82% and 39.55%, χ^2^ = 12.24, *p* = 0.02). PrEP-naïve and unwilling group were significantly more likely to reside in rural areas (rural: 38.72% vs. 14.29% and 14.18%, χ^2^ = 82.78, *p* < 0.001) compared to PrEP-naïve and unwilling group and current/former user group and had significantly lower educational levels (high school:34.21% vs. 67.48% and 57.46%, χ^2^ = 153.44, *p* < 0.001), and had significantly lower income levels (6001–9000 RMB:15.04% vs. 30.48% and 32.84%, χ^2^ = 166.82, *p* < 0.001). (Table 1)

**Fig. 1:**
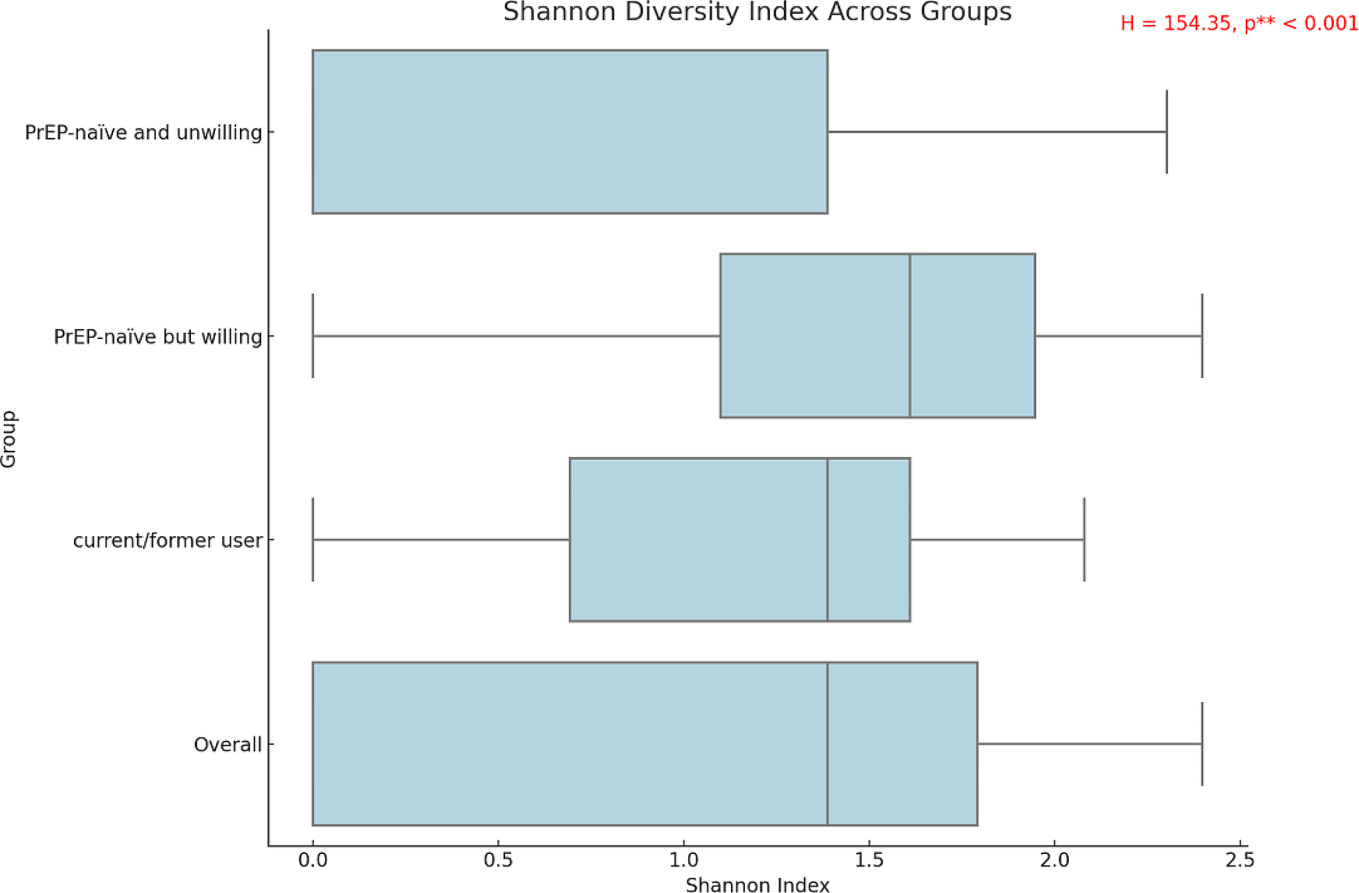
Differences in Shannon index among the three groups. *Note* p**: there is a statistical difference between the three groups; H:Kruskal-Wallis H test

PrEP-naïve but willing group had the highest STI testing rate in the last three months compared to PrEP-naïve and unwilling group and current/former user group (73.06% vs. 66.17% and 66.92%, χ^2^ = 6.33, *p* = 0.04). Additionally, they also had a higher median Shannon index compared to the other two groups (1.61(1.10,1.95) vs. 0(0,1.39) and 1.39(0.69,1.61), $H$= 154.35, *p* < 0.001), as shown in Fig. 1. The current/former PrEP user group exhibited the highest awareness rates for PrEP (90.77% vs. 86.12% and 50.75%, χ^2^ = 187.71, *p* < 0.001) as well as the types: oral PrEP (86.92% vs. 84.76% and 48.12%, χ^2^ = 156.44, *p* < 0.001), 2-1-1 PrEP (88.46% vs. 67.48% and 46.24%, χ^2^ = 72.72, *p* < 0.001), and long-acting injectable PrEP (46.15% vs. 42.72% and 23.31%, χ^2^ = 34.08, *p* < 0.001) and reported a higher perceived risk of infection (scores of 4–6: 39.23% vs. 28.03% and 22.93%, χ^2^ = 15.11, *p* = 0.004). Despite this, they reported less frequent condom use during anal intercourse over the past three months (Use condoms every time: 51.54% vs. 68.30% and 79.32%, χ^2^ = 52.01, *p* < 0.001) and had a higher prevalence of chemsex (20.00% vs. 11.97% and 6.77%, χ^2^ = 14.92, *p* < 0.001). (Table 2)

**Table 2:** Differences in HIV and STI testings, PrEP awareness, and related behaviors among three PrEP usage groups

Category	PrEP-naïve and unwilling group(N = 266)	PrEP-naïve but willing group(N = 735)	Current/former user group(N = 130)	Chi-Square	*P*-value
**Date of last HIV testing**				0.68	0.711
Within 3 months	193 (72.56%)	538 (73.20%)	95 (73.08%)		
Beyond 3 months	61 (22.93%)	160 (21.77%)	24 (18.46%)		
**Last time testing for STIs**				6.33	0.042*
Within 3 months	176 (66.17%)	537 (73.06%)	87 (66.92%)		
Beyond 3 months	86 (32.33%)	190 (25.85%)	24 (18.46%)		
**Ever heard of HIV Prep**				187.71	<0.001**
Yes	135 (50.75%)	633 (86.12%)	118 (90.77%)		
No	118 (44.36%)	62 (8.43%)	12 (9.23%)		
**Ever heard of daily oral Prep**				156.44	<0.001**
Yes	128 (48.12%)	623 (84.76%)	113 (86.92%)		
No	134 (50.38%)	104 (14.15%)	17 (13.08%)		
**Ever heard of PrEP (2-1-1)**				72.72	<0.001**
Yes	123 (46.24%)	496 (67.48%)	115 (88.46%)		
No	139 (52.26%)	231 (31.43%)	15 (11.54%)		
**Ever heard of long-acting injectable PrEP**				34.08	<0.001**
Yes	62 (23.31%)	314 (42.72%)	60 (46.15%)		
No	200 (75.19%)	413 (56.19%)	70 (53.85%)		
**Do you think you are likely to be infected with HIV**				15.11	0.004**
0–3	194 (72.93%)	503 (68.44%)	71 (54.62%)		
4–6	61 (22.93%)	206 (28.03%)	51 (39.23%)		
7–10	10 (3.76%)	29 (3.95%)	9 (6.92%)		
**Condom use where active anal intercourse occurred in the past three months**				57.01	<0.001**
Every time	184 (69.17%)	467 (63.54%)	50 (38.46%)		
Sometimes	16 (6.02%)	110 (14.97%)	40 (30.77%)		
Never use	11 (4.14%)	23 (3.13%)	1 (0.77%)		
**Condom use where passive anal intercourse occurred in the past three months**				52.01	<0.001**
Every time	211 (79.32%)	502 (68.30%)	67 (51.54%)		
Sometimes	13 (4.89%)	126 (17.14%)	38 (29.23%)		
Never use	14 (5.26%)	17 (2.31%)	3 (2.31%)		
**Drug use in the last sexual activity(chemsex)**				14.92	<0.001**
Yes	18 (6.77%)	88 (11.97%)	26 (20.00%)		
No	247 (92.86%)	647 (88.03%)	104 (80.00%)		

*Note* Do you think you are likely to be infected with HIV: The chart is based on participants’ assessment of their likelihood of contracting HIV. Scores range from 0 (believing there’s no likelihood at all) to 10 (believing it’s highly likely); * : statistical significance at *p* < 0.05 level; **: higher level of statistical significance at *p* < 0.01

### Factors Associated with PrEP Usage in MSM

**Fig. 2:**
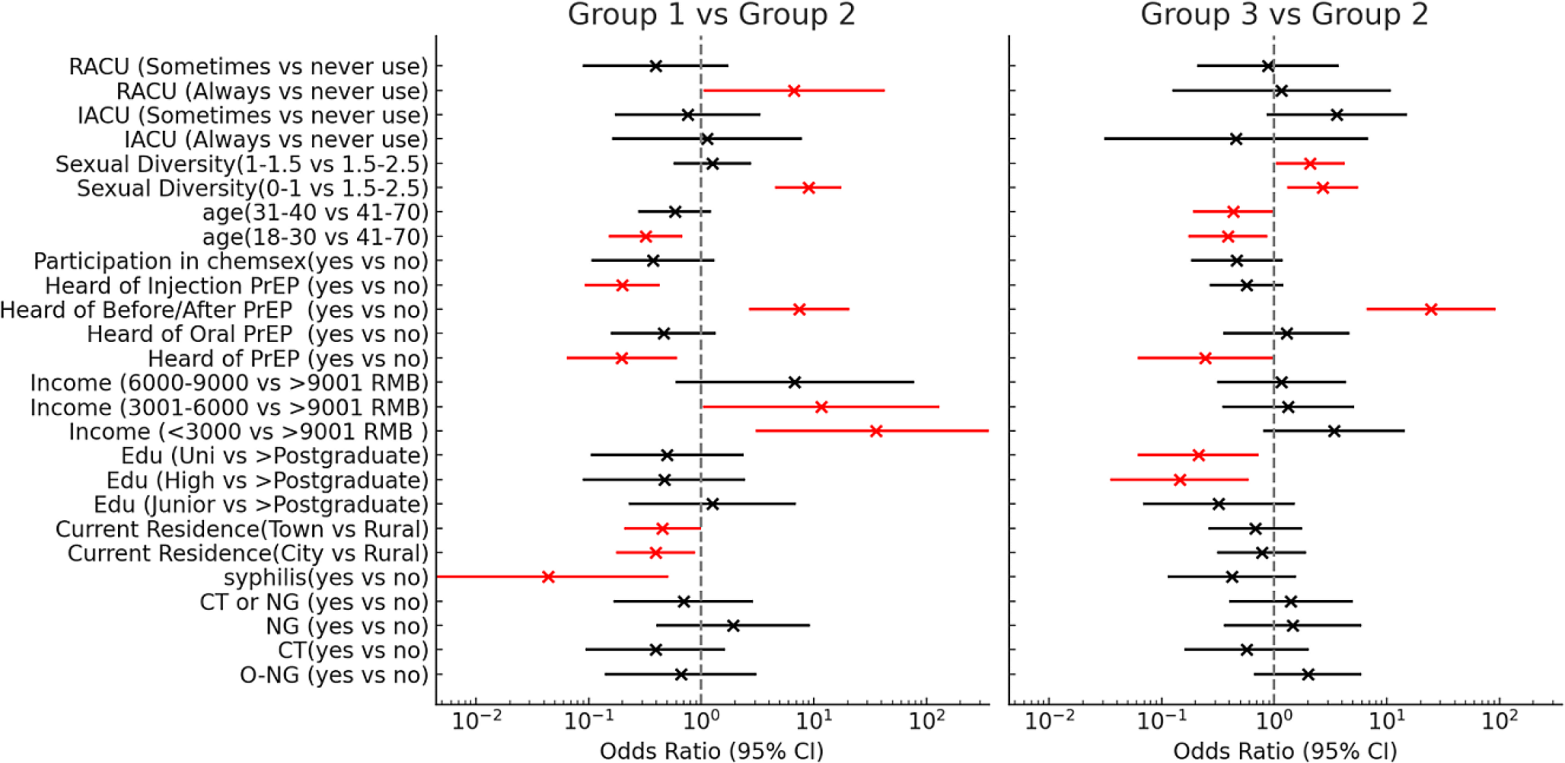
Factors influencing PrEP usage among MSM from a multivariable regression. *Note* Group 1 : the PrEP-naïve and unwilling group; Group 2 : PrEP-naïve but willing group; Group 3 :Current/former user group; ‘O-NG’ : oropharyngeal gonorrhea; ‘CT’ :chlamydia infection; ‘NG’ : gonorrhea infection; ‘IACU (Always vs never use)’ :consistent versus no condom use during insertive anal intercourse in the last three months; ‘RACU (Always vs never use)’ : consistent versus no condom use during receptive anal intercourse in the last three months; Red coloration: data points where the p-value is less than 0.05, indicating statistical significance

In comparing PrEP-naïve but willing group and current/former users, age was a significant predictor. Younger MSM aged 18–30 and 31–40 exhibited lower odds of PrEP usage (OR(95%CI): 0.34(0.18–0.85), *p* = 0.02; 0.43(0.20–0.96), *p* = 0.04). Education level also impacted PrEP usage, with MSM having lower educational attainment showing decreased odds of PrEP usage (high school/vocational: OR(95%CI):0.15(0.04–0.58), *p* = 0.01; associate degree: OR(95%CI):0.21 (0.06–0.71), *p* = 0.01). Awareness of the 2-1-1 dosing for PrEP was found to increase the likelihood of PrEP usage (OR(95%CI):24.78 (6.83–89.95), *p* < 0.001), while general PrEP awareness surprisingly decreased it (OR(95%CI):0.24 (0.06–0.94), *p* = 0.04). Moreover, sexual diversity, as reflected by the Shannon index, also played a role in PrEP usage, as MSM with an Index of 0–1 and 1-1.5 were more likely to use PrEP (OR(95%CI):2.69 (1.35–5.38), *p* = 0.01; OR = 2.09, *p* = 0.03).(Fig. 2).

When the PrEP-naïve but willing group was compared to the PrEP-naïve and unwilling group, Attitudes towards PrEP were found to be influenced by various factors, including a diagnosis of syphilis and the location of residence. Participants who had been diagnosed with syphilis were less likely to reject using PrEP (OR(95%CI):0.04 (0.004–0.49), *p* = 0.01), as were those living in cities or towns (OR(95%CI):0.40 (0.18–0.86), *p* = 0.02; OR = 0.45 (0.21–0.98), *p* = 0.04). Lower income levels (< 3000 RMB: OR(95%CI):35.78 (3.15-406.26), *p* < 0.01; 3000–6000 RMB: OR(95%CI):11.64 (1.07–127.30), *p* = 0.04) increased the likelihood of PrEP nonuse. Overall, having knowledge about PrEP reduced nonuse (HIV PrEP: OR(95%CI):0.20 (0.07–0.59), *p* < 0.01; 2-monthly injections: OR(95%CI):0.20 (0.10–0.42), *p* < 0.001), but increased awareness of the 2-1-1 dosing for PrEP decreased the willingness of usage (OR(95%CI):7.39(2.72–20.03), *p* < 0.001). A Shannon Index score of 0–1 also increased avoidance (OR(95%CI):8.94(4.67–17.10), *p* < 0.001), while being in the 18–30 age group and consistently using condoms increased disinclination towards PrEP (OR(95%CI):0.32 (0.16–0.66), *p* < 0.01; OR = 6.69 (1.08–41.42), *p* = 0.04). When comparing current/former PrEP users to PrEP-naïve and unwilling group, awareness of injectable PrEP significantly increased the likelihood of its use (OR(95%CI): 0.35 (0.14–0.86), *p* = 0.02). In contrast, a lower Sexual Diversity score markedly decreased willingness to use PrEP (OR(95%CI): 3.32 (1.43–7.71), p = < 0.01). (Fig. 2)

### STI Prevalence Among Three PrEP Use Groups

Current/former user group had the highest prevalence of O-NG infection(14.39% versus 9.68% and 5.80%, χ^2^ = 9.83, *p* < 0.01) and NG infection(20.86% versus 17.17% and 8.37%, χ^2^ = 13.99,*p* < 0.001), compared with both the PrEP-naïve but willing group and the PrEP-naïve and unwilling group. The PrEP-naïve but willing group had the highest prevalence of syphilis(7.49% versus 6.47% and 2.54%, χ^2^ = 6.14,*p* = 0.046) and CT or NG infections(31.08% versus 28.78% and 16.03%, χ^2^ = 14.14,*p* < 0.001), compared to current/former users and the PrEP-naïve and unwilling group. No significant differences were observed for other infection sites and diseases among the groups, as indicated in Table 3.

**Table 3:** STI prevalence at various infection sites among three PrEP usage groups

Infection Sites & PrEP Use Status	PrEP-naïve and unwilling group(N = 266)		PrEP-naïve but willing group(N = 735)		Current/former user group(N = 130)		Chi-Square	P
	Positive	Negative	Positive	Negative	Positive	Negative		
A-CT	25 (9.09%)	241 (90.91%)	94 (14.67%)	641 (85.33%)	13 (9.35%)	117 (90.65%)	2.58	0.276
A-NG	8 (2.91%)	258 (97.09%)	46 (7.18%)	689 (92.82%)	8 (5.76%)	122 (94.24%)	4.11	0.128
O-CT	2 (0.73%)	264 (99.27%)	9 (1.40%)	726 (98.60%)	1 (0.72%)	129 (99.28%)	0.53	0.765
O-NG	16 (5.80%)	250 (94.20%)	62 (9.68%)	673 (90.32%)	20 (14.39%)	110 (85.61%)	9.83	0.007**
U-CT	4 (1.45%)	262 (98.55%)	12 (1.87%)	723 (98.13%)	1 (0.72%)	129 (99.28%)	0.56	0.757
U-NG	0 (0%)	266 (100%)	15 (2.34%)	720 (97.66%)	3 (2.16%)	127 (97.84%)	5.68	0.059
HIV	7 (2.54%)	259 (97.46%)	28 (4.37%)	707 (95.63%)	4 (2.88%)	126 (97.12%)	0.87	0.646
Syphilis	7 (2.54%)	259 (97.46%)	48 (7.49%)	678 (92.51%)	9 (6.47%)	121 (93.53%)	6.14	0.046*
CT	28 (10.18%)	238 (89.82%)	110 (17.17%)	625 (82.83%)	15 (10.79%)	115 (89.21%)	3.79	0.150
NG	23 (8.37%)	243 (91.63%)	110 (17.17%)	625 (82.83%)	29 (20.86%)	101 (79.14%)	13.99	<0.001**
CT or NG	44 (16.03%)	222 (83.97%)	199 (31.08%)	536 (68.92%)	40 (28.78%)	90(71.22%)	14.14	<0.001**

*Note* CT: Chlamydia trachomatis infection; NG: Neisseria gonorrhoeae infection; A: Anal. O: Oral. U: Urethral. CT or NG: participants infected with Chlamydia trachomatis, Neisseria gonorrhoeae, or both; *: statistical significance at *p* < 0.05 level; **: higher level of statistical significance at *p* < 0.01

## Discussion

Nearly 80% of our study’s participants have never used PrEP. Among these, two-thirds are willing to try it, and one-third are not.The PrEP-naïve but willing group, primarily younger MSM, exhibited the highest rates of STI testing and the greatest diversity in sexual behavior. This group also recorded the highest rates of CT or NG infections and syphilis. Conversely, the PrEP-naïve and unwilling group, typically from rural areas with lower education and income, were the most hesitant towards PrEP. The current/former user group showed the highest prevalence of O-NG and NG infections and least frequent condom use. Moreover, we found that Age, education, sexual behavior diversity (Shannon index), and knowledge of the 2-1-1 PrEP dosing regimen emerged as significant influences on PrEP adoption.

The proportion of PrEP-naïve but willing MSM in Xi’an aligns with findings from Guangzhou, Shanghai, Beijing, Shenyang, Chongqing, and Sichuan, where interest ranges from 63–89% [5, 43–48]. The PrEP-naïve but willing group mainly included younger, urban residents with higher education and income levels. These demographic characteristics suggest that is more easily educated about sexual health and more inclined to accept PrEP, benefiting from improved accessibility and awareness opportunities [49–53]. Moreover, better education is associated with greater awareness of PrEP’s advantages. Meanwhile, income level not only affects PrEP affordability but also indicates engagement with proactive PrEP communities, aligning with findings from PrEP cost-related adoption studies [44, 46, 48].

Multivariable logistic regression pinpointed factors like age, education, residential location, income, PrEP perception, condom use, and syphilis infection as influencers of PrEP status, consistent with previous studies [5, 23, 54]. Sexual diversity impacts PrEP use and willingness differently across groups. PrEP-naïve and unwilling group see low diversity as a reason for their reluctance. Low sexual diversity may be interpreted as low-risk behavior, leading people to be unwilling to consider using PrEP. In contrast, this finding contradicts our other results regarding actual current/former user group compared to PrEP-naïve but willing group. This difference may be related to the self-perception of HIV risk. People who believe that they are at a higher risk of HIV infection may still engage in high-risk sexual behaviors, despite limited sexual diversity, and therefore have a greater inclination to use PrEP. Meanwhile, men with high sexual diversity who are potential PrEP users often receive risk-related medical advice following STI infections. This advice boosts their willingness to use PrEP, though they may not yet have taken action to start it. Individuals informed about sex-driven PrEP usage were more inclined to use it. This aligns with a study in China where MSM preferred such PrEP approaches [5]. However, in comparisons between the PrEP-naïve but willing group and the PrEP-naïve and unwilling group, those informed about sex-driven PrEP usage showed less likelihood of using it. This reluctance may stem from worries over the inconvenience or the risk of forgetting to take the medication according to the 2-1-1 dosing regimen’s specific timing around sexual activity. some may not see themselves as part of a high-risk group, further reducing their interest in using PrEP.

Older individuals (aged 40–70) frequently transitioned from willingness to actual PrEP utilization, which is possibly due to the prevalence of urban living offering better healthcare access and targeted initiatives. Younger ones might not use PrEP much because they know less about getting it, even though they’re interested. That substantiated by a study highlighting their inclination yet limited understanding and access knowledge [55]. People with syphilis showed a greater inclination towards PrEP, mirroring a Chinese study’s observation linking STIs with amplified sexual health awareness and PrEP adoption [38].

Current/former user group and PrEP-naïve but willing gourp showed higher rates of STIs, including gonorrhea, chlamydia, and syphilis. One potential reason for the heightened STI infection rate among PrEP-naïve but willing group could be linked to their higher sexual acts diversity. Despite this group reporting a higher rate of condom use compared to current/former users, they engage in more complex sexual activities during singular encounters. This intricacy in their sexual behaviors might contribute to the increased infection rate. These findings are consistent with previous research, which has found that individuals with STI infections are more likely to be willing to use or actually use PrEP. This pattern resonates with the observations made by Traeger, which highlighted increased STI rates post-initiation of PrEP, hinting at elevated risk behaviors [56]. Moreover, Former/Current PrEP users reported inconsistent condom use and increased chemsex, suggesting a potential perception of reduced HIV risk, which in turn may raise the risk of orther STI infections. These findings mirror studies that link PrEP usage with riskier sexual behaviors [5, 38]. Similarly, PrEP-naïve but willing group show a significant correlation between STI testing and diverse sexual behaviors, indicating a history of STIs or engagement in riskier activities. This finding aligns with a study conducted in California [57].

Some studies did not find a statistically significant difference in STI prevalence between PrEP users and non-users, while others found an association between PrEP use and increased STI risk [58–61]. One potential reason for these discrepancies is the presence of two distinct high-risk groups for STI infections within the MSM population: Former/Current PrEP user group and PrEP-naïve but willing group. By solely distinguishing between PrEP users and non-users, studies may overlook the high-risk of STI infections faced by the PrEP-naïve but willing gourp. Considering that individuals willing to use PrEP, even if they have not started using it, may engage in similar high-risk sexual behaviors or have a history of STI infections as those who are already using PrEP [62, 63].

Despite the widespread willingness among the MSM population to use PrEP, there are still many individuals who have not yet started using PrEP. Given the heightened risk of HIV transmission due to STIs, it’s crucial to encourage PrEP uptake. This finding not only creates an environment for better dissemination of education and recommendations about PrEP to those willing but not yet using it but also ensures control of concurrent STI infections, thus minimizing potential HIV infection rates.

Several limitations should be noted in this study. Firstly, the study design is cross-sectional, which prevents us from establishing causality between variables. While we can observe associations, we cannot definitively conclude that one variable causes another. Secondly, there is a potential limitation of selection bias in our sample. Secondly, our sampling method, which blended simple random and snowball techniques, may introduce selection bias due to the predominant online recruitment. This approach might not capture the broader MSM population, particularly those offline, potentially skewing our findings and limiting generalizability. Thirdly, due to the self-reporting nature of the study, there may be recall bias and social desirability bias. Participants might not accurately remember or may be inclined to report behaviors in a socially acceptable manner, which could affect the validity of our findings. Despite these limitations, our research explores the impacts of various factors during a last sexual activity on PrEP usage and willingness, offering a profound understanding that is often overlooked in similar studies.

## Conclusion

‘PrEP-naïve but willing’ participants consistently demonstrate high-risk of sexual behaviour, increased STI testing rates and a higher sexual act diversity index, whereas PrEP users demonstrate the highest STI prevalence. Our study results also highlight that individuals residing in urban areas, who are younger, well-educated, and have higher incomes are more likely to be willing or actually use PrEP.
